# Effect of case management on viral load in adult clients enrolled due to non-suppression in Capricorn District, South Africa: A case control study

**DOI:** 10.1371/journal.pone.0317015

**Published:** 2025-01-06

**Authors:** Molebatsi Moholola, Kate Rees, Nthabiseng Motsoane, Ntsetse Kgopong, Chipo Mutyambizi

**Affiliations:** 1 Anova Health Institute, Capricorn, South Africa; 2 Department of Community Health, School of Public Health, University of the Witwatersrand, Johannesburg, South Africa; 3 Anova Health Institute, Johannesburg, South Africa; Ruedi Luethy Foundation - Zimbabwe, ZIMBABWE

## Abstract

**Background:**

Case management is a structured, client centered approach that incorporates various strategies such as employing lay counsellors to provide psychosocial and adherence support to strengthen antiretroviral (ART) adherence, improve retention in care and viral load (VL) suppression. This study aimed to evaluate the effects of case management on VL in clients enrolled due to non-suppression (> = 50 copies/ml) in Capricorn District, Limpopo Province.

**Methods:**

We conducted a case control study using two datasets (1) cases were selected from case management data collected from June 2021 to November 2022 at 35 facilities and captured on the REDCap system. (2) controls were identified from TIER.Net data from facilities where case management is not available and with at least two VLs on record since June 2021. Our study was restricted to clients with an unsuppressed (> = 50 copies) VL at enrollment, over the age of 18 years and excluded clients with a missing VL at enrollment. Using similar age, gender, and VL characteristics, an equal number of clients not receiving case management was randomly sampled from the TIER.Net data. Descriptive and multivariate logistic regression analysis were used to determine the factors associated with viral suppression.

**Results:**

Our final study sample consisted of 3 256 clients, half of which received case management (N = 1 628), 1084 (33%) with a first VL in study of 50–399 copies/ml, 404 (12%) 400–999 copies/ml and 1768 (54%) >1000 copies/ml. Post case management intervention results showed that 49% had a VL below 50 copies/ml amongst those receiving case management and 44% among those who did not receive case management. In the adjusted model we found that case management (Odds ratio [OR] 1.25; 95% Confidence Interval [CI] 1.08–1.44) versus no case management, 35–54 years old (1.43; 1.07–1.91) and 55+ year old (1.88; 1.35–2.61) versus 18–24-year-old increased odds of VL suppression whilst being male (0.72; 0.61–0.84) versus being female has decreased odd of VL suppression.

**Conclusion:**

Close to half of the clients had a VL below 50 copies/ml after case management. Factors that increased the odds of VL suppression were case management and older age, whilst being male was associated with reduced odds of VL suppression. Differentiated services for virally unsuppressed clients would be helpful for men. Case management was associated with viral suppression in those with a starting VL > 1000 copies/ml and not for those starting with low level viremia (50–999 copies/ml).

## Introduction

Human Immunodeficiency Virus (HIV) remains one of the world’s significant public health challenges. In 2020, the global HIV response shifted from the 90-90-90 targets to the 95-95-95 targets, aimed at ensuring that 95% of people living with HIV (PLHIV) know their status, 95% of those diagnosed receive antiretroviral therapy (ART) and 95% of those on ART achieve viral suppression [1]. In March 2023 the South African HIV care cascade was at 94-77-92 [2]. ART coverage and viral suppression are still below target and when measured against the 7.9 million PLHIV in South Africa, 74% are on ART and 53% are virally suppressed [3, 4].

Various thresholds have been applied when estimating viral suppression for different settings [5, 6]. South African National Guidelines define viral load (VL) suppression as <50copies/ml [7] whilst the World Health Organisation (WHO) uses a threshold of <1000 copies/ml [8]. Unsuppressed VL is associated with HIV disease progression, increased morbidity, mortality and increased HIV transmission risk [9]. Especially in the era of Tenofovir, Lamivudine and Dolutegravir (TLD), poor adherence is the most common cause of an unsuppressed VL [10]. Although access to ART has increased in South Africa, one of the vital concerns is treatment adherence, and supporting improved adherence remains a challenge for healthcare providers [11]. Barriers to adherence and retention are numerous and include patient-related barriers such as mental health and intimate partner violence [12], as well as healthcare barriers such as overburdened health facilities and lack of empathy from healthcare workers, and social factors such as stigma and lack of support from family and friends [13, 14].

To address poor adherence in people with a high VL, the WHO recommends the use of enhanced adherence counselling (EAC) [9]. The South African ART clinical guidelines recommend EAC for clients with an elevated VL (>50 copies/ml) experiencing challenges with taking/remembering to take treatment [7]. Case management is one of the mechanisms through which EAC is implemented. The goals of EAC are centered around supporting individuals to achieve and maintain optimal adherence to their ART medication. This is achieved through an assessment of clients’ barriers to adherence and retention and developing strategies to overcome these [7]. Two sessions are recommended with the first session provided to patients struggling with adherence. Persistent non-adherent clients are referred for a second session only if their second VL, taken 3 months after the first session is >50 copies/ml [15]. Previous studies have shown the benefits of EAC [16–19]. In Zambia, about 61% of clients who underwent EAC were virally suppressed at the end of 3months [16]. Similarly, Diress et al (2020) showed about 66% of clients enrolled on EAC achieved viral suppression [17].

Studies on the VL outcomes of case management for PLHIV in South Africa are scarce. Our study sought to evaluate changes in VL amongst unsuppressed clients enrolled in case management and to determine the impact of case management on VL suppression in Capricorn District, Limpopo.

## Methods

### Description of intervention: Case management in Capricorn District

Anova Health Institute, a Presidents Emergency Plan for AIDS Relief (PEPFAR)/ United States Agency for International Development (USAID) district support partner for the Department of Health in South Africa, supported the Limpopo Department of Health to implement a case management model in Capricorn District. The model was introduced in selected facilities in December 2020, and an electronic record keeping system implemented in June 2021. The model uses a team of case managers, also known as retention counsellors, who are lay counsellors trained on national adherence guidelines (including Fast track initiation counselling and—EAC) and motivational interviewing [20]. Retention counsellors are based at high volume health facilities to provide patient-centered supportive services.

The programme targets clients living with HIV who are at high risk of poorer treatment outcomes such as those who are newly diagnosed, have been disengaged and are now re-engaging, and those who are virally unsuppressed (> 50 copies/ml). Anova’s case management programme aims to deliver client-centered services to improve treatment adherence and retention in care. Clients are recruited into the programme for 180 days of comprehensive adherence support and follow-up engagements through referrals from clinicians, non-governmental organizations (NGOs), and health talks [20]. Retention counsellors work with clients to plan their adherence support sessions, based on clients’ needs, preferences and availability. During the sessions, barriers to adherence are assessed, strategies to overcome these barriers are developed and referrals for population specific services such as postnatal clubs are facilitated. Case management engagements can take place individually or in groups via telephone, face-to-face, WhatsApp or SMS, covering treatment literacy, medication side effects, treatment barriers, disclosure, and safe sex. B-OK counselling beads bottles are used to demonstrate VL and amplify the concept that undetectable is equal to untransmittable [21]. Education flipcharts and videos are also used during treatment literacy sessions. All action points discussed in the sessions are documented in the patient’s file and fast track initiation and EAC registers. Telephonic contact to follow up on previous discussions is provided every two weeks. Weekly adherence support messages are sent to the client and appointment reminders via telephone call two to three days before the client’s facility visit. Case managed clients who miss their appointments are also traced within 24hours.

Clients undergo viral load collection after three months of case management. Those who are unsuppressed continue case management and those who are suppressed move to multi-month dispensing. Retention counsellors are supported by adherence officers. At the time of the study, 35 (33%) of the 105 facilities in Capricorn District were implementing the case management model. These facilities were selected for case management implementation because of their high number of ART clients.

### Study design and setting

We conducted a case control study using two datasets (1) Cases were taken from data on case management captured on the REDCap system (further referred to as REDCap case management data). (2) The control group was taken from TIER.Net data exported on 18 January 2023. TIER.Net is an electronic ART database developed by the University of Cape Town’s Centre for Infectious Disease Epidemiology and Research [22] to document demographic characteristics, clinical care and treatment outcomes over time for people living with HIV in South Africa. It is used in the majority of South Africa’s public sector facilities.

The study was conducted in Capricorn, one of the 5 districts in Limpopo Province, South Africa. The Naomi Model estimates an HIV prevalence for 15–49-year age group in Limpopo Province of 17.2%, lower than the national prevalence of 18.4%, and there are more than 430 000 people on ART [23]. Capricorn District is made up of 4 local municipalities (Blouberg, Lepelle-Nkumpi, Molemole and Polokwane) and has a population of approximately 1.3 million people [24]. Health care is provided via a mix of private and public health facilities. The district is predominantly rural, and the trade sector, finance and community services contribute the most to the district’s economy.

### Data

#### 1. REDCap case management data–Cases

Data is collected at case management enrollment, and each engagement. The data was anonymized at the point of downloading from REDCap on 6 December 2022. For this analysis, variables extracted from REDCap included age, gender, ART enrollment status (unsuppressed), VL at enrollment, and the VL taken after enrollment.

### Inclusion and exclusion criteria

**Fig 1:**
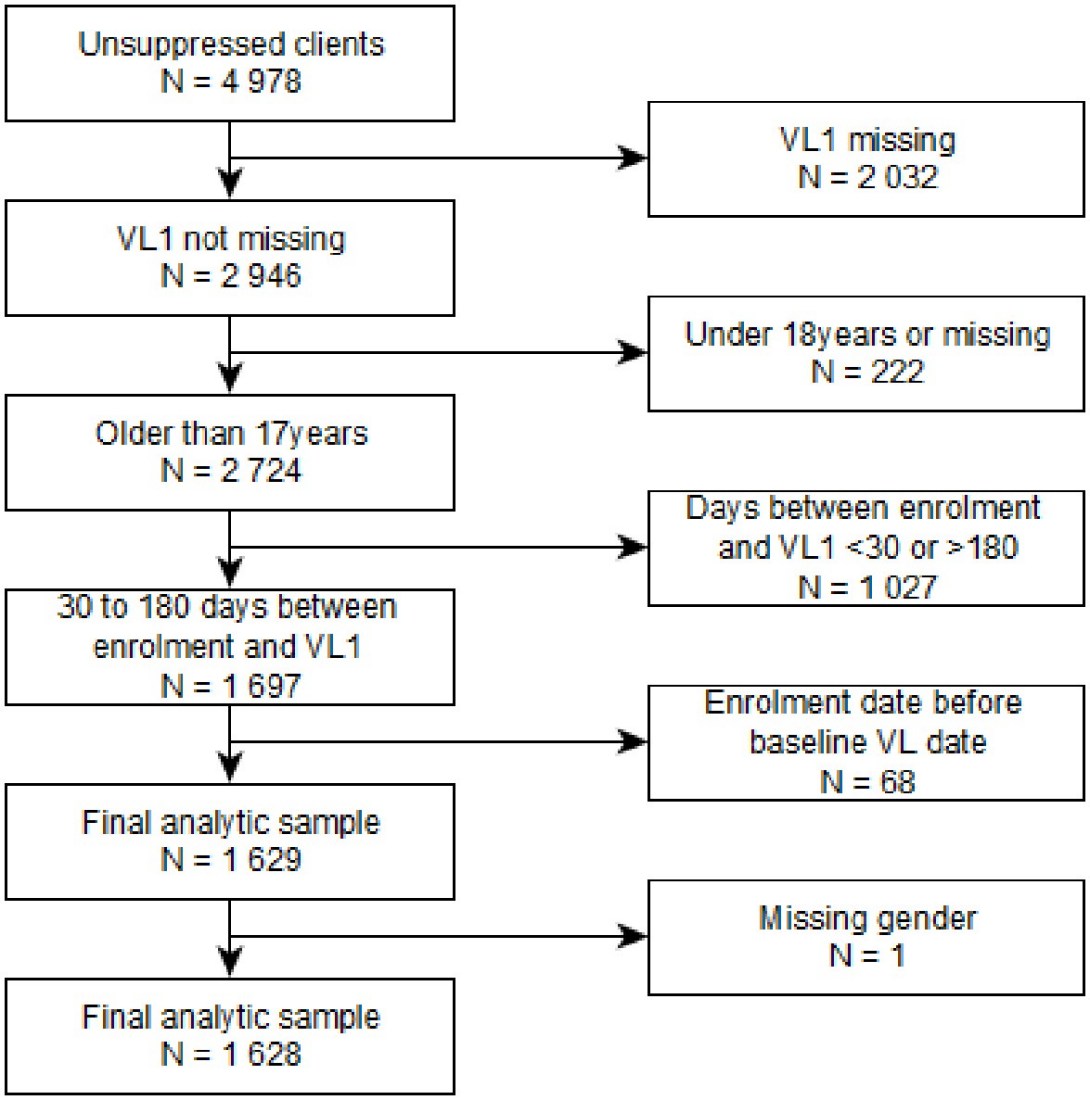
REDCap data flow chart for selecting final analytic sample.

The case management dataset consisted of 4978 clients with an unsuppressed VL taken before enrollment into case management (further referred to as first VL in study). VL suppression was defined as a VL < 50 copies/ml as per South African guidelines [7]. Of these unsuppressed clients 53% (2634/4978) had a VL of 50 to 999 copies/ml. Our analysis excluded those without a VL on record AFTER undergoing case management (further referred to as second VL in study). To restrict our analysis to the adult sample we excluded those below the age of 18 years (n = 222). National ART guidelines require that clients with an unsuppressed VL have a repeat VL after 3 months. We restricted our analysis to those who had a second VL in study date between 30 to 180 days after their enrollment date. We also excluded those that had a case management enrollment date before the first VL date and those missing gender. Our final analytic sample was 1 628 records (see Fig 1).

#### 1. TIER.Net data- controls

**Fig 2:**
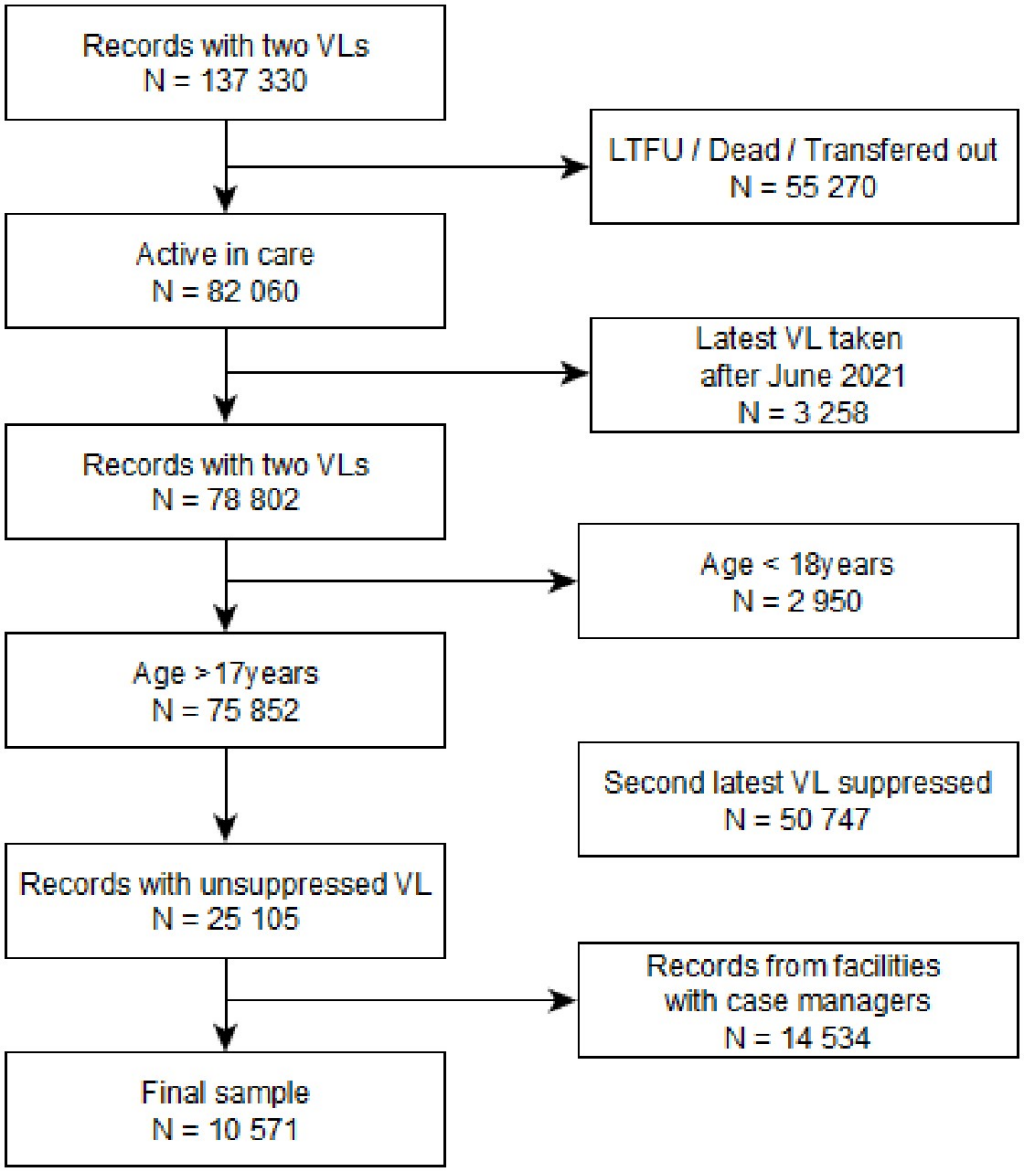
Facility level data flow chart for selecting final analytic sample.

We analysed routinely available, anonymised TIER.Net data, extracted from TIER.Net on 18 January 2023. Data was fully anonymised before author access. We extracted all records containing at least two VL counts (N = 137 330) after June 2021 (see Fig 2) and restricted our VL analysis to the first 2 VL results. We further restricted our analysis to those whose first VL in study was unsuppressed (> = 50 copies). We excluded those below the age of 18 years and restricted the sample to only those records from facilities without retention counsellors. The final sample of 10 571 was then used to randomly select controls.

### Control selection

The following steps were then followed in STATA to select a sample of records from those receiving no case management (TIER.Net data), that had similar characteristics to those receiving case management (REDCap data). (1) We created groups with similar gender, age category (19 to 24 years, 25 to 34 years, 35 to 54 years and 55+ years) and first VL in study (50 to 399 copies/ml, 400 to 999 copies/ml and > 1000 copies/ml) (30 groups created in total). We then (2) counted number of clients receiving case management in each group in REDCap data and (3) randomly sampled an equal number of clients not receiving case management from each group in TIER.Net data. The final sample used in this analysis was 3 256 records (see S1 Dataset).

### Ethical approval

Ethical approval was obtained from the Human Sciences Research Council Research Ethics Committee (REC 3/22/08/18). This study used anonymised REDCap and TIER.Net data which is routinely collected at healthcare facilities for monitoring purposes, therefore individual consent was not required, as no data collection was performed and no client files were accessed.

### Measures

The main outcome is second VL in study. This was coded as a binary variable and took on the value of 0 if client is unsuppressed (> = 50 copies/ml) and 1 if client is suppressed (<50 copies/ml).

Our intervention variable was case management. For other covariates we included age, gender, and number of years on ART. Further details on these measures are provided in Table 1.

**Table 1:** Variable included in analysis.

Variable	Definition	Category
**Outcome variable**		
Second VL in study suppression	VL taken between 30 to 180 days after case management enrollment is <50copies/ml	0 = unsuppressed, 1 = suppressed
**Covariates**		
Age	Age of client	1 = 18 to 24 years, 2 = 25 to 34 years, 3 = 35 to 54 years, 4 = 55years and over
Gender	Gender of client	1 = female, 2 = male
ART years	How many years has the client been on ART	0 = less than 1 year, 1 = 1 to 3 years, 2 = 3 to 10 years, 3 = over 10 years
**Intervention variable**		
Case management	Did the client receive case management	0 –no case management, 1 –case management

### Data analysis

Statistical analysis was conducted in STATA 14. In reporting the descriptive statistics proportions were used for categorical variables. We made use of multivariate logistic regression analysis to determine the factors associated with viral suppression. The impact of receiving case management on viral suppression was assessed using both bivariate and multivariate logistic regression. We also conducted a multivariate logistic regression analysis stratified by first VL in study categorised as follows: 1–50–999 copies/ml (low level viremia), 2–1000 to 9999 copies/ml and 3—>9999 copies/ml.

## Results

The characteristics of the study sample are shown in Table 2. Our study sample consisted of 3 256 records, half of which received case management (N = 1 628). The majority (54%, 1768/3256) of the patients in our study sample had a first VL in study > 1000 copies/ml and 46% (1488/3256) had low level viremia (50 to 999copies/ml). 69% of the study sample were females (2246/3256) and 57% (1866/3256) were between the ages of 35 to 54 years. Approximately 61% of clients have been on ART for between 3 to 10 years (see Table 2).

**Table 2:** Characteristics of those receiving case management versus those not receiving case management.

Variable	Total analytic sample N = 3 256		Case management N = 1 628		No Case management N = 1 628	
	N	%	N	%	N	%
Case management						
No	1628	50				
Yes	1628	50				
Enrollment viral load						
50 to 399	1084	33	542	33	542	33
400 to 999	404	12	202	12	202	12
> 1000	1768	54	884	54	884	54
Gender						
Female	2246	69	1123	69	1123	69
Male	1010	31	505	31	505	31
Age category						
18–24	222	7	111	7	111	7
25–34	666	21	333	20	333	21
35–54	1866	57	933	57	933	57
55+	502	15	251	15	251	15
ART years						
<6 months	43	1	43	3		
6–12 months	134	4	65	4	69	4
1 to 3 years	510	16	295	18	215	13
3 to 10 years	1967	61	968	60	999	61
over ten years	583	18	238	15	345	21

**Fig 3:**
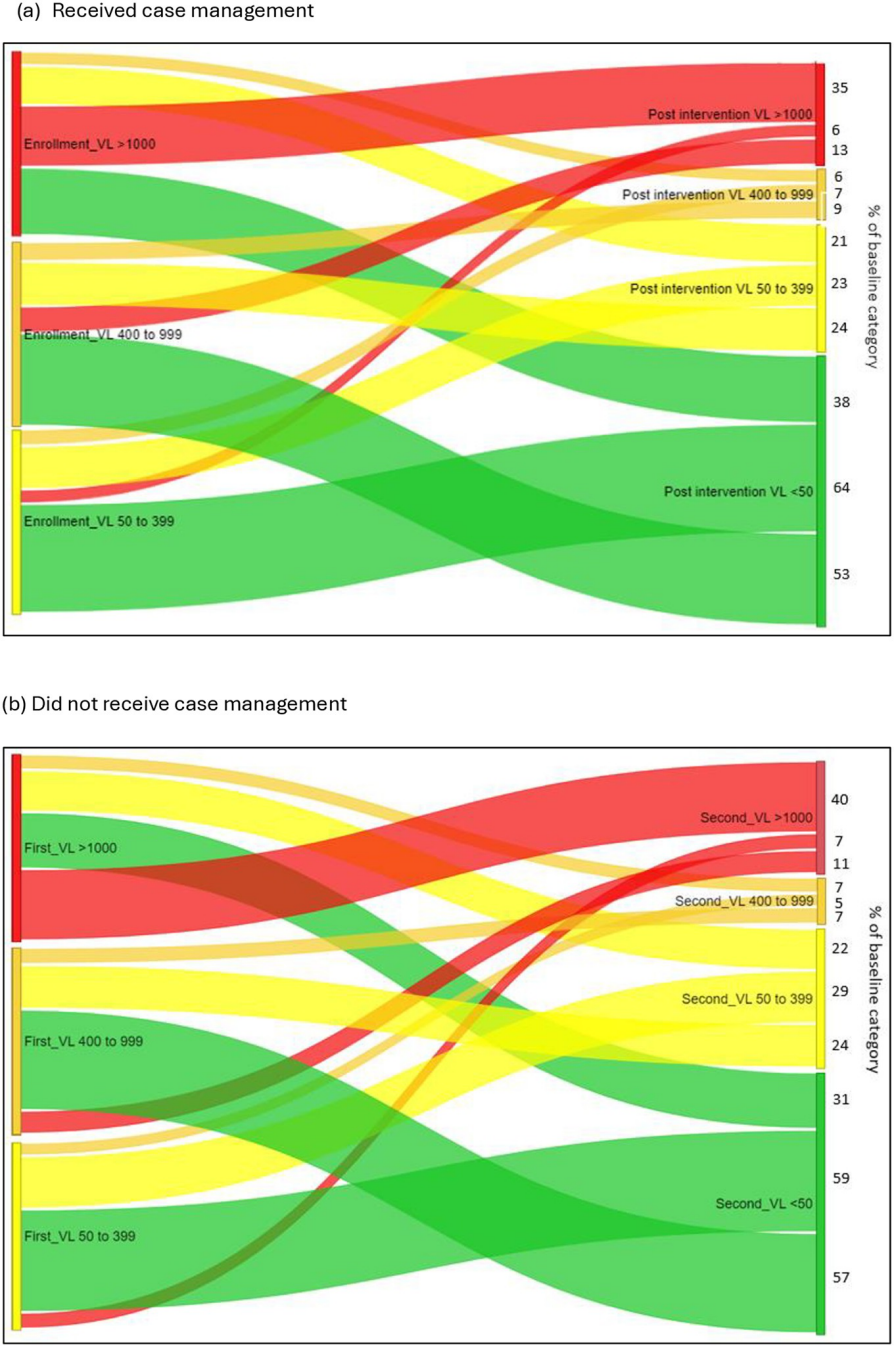
Changes in VL for clients who received and did not receive case management. (a) Received case management, (b) Did not received case management.

Amongst clients who received case management second VL was below 50 copies/ml in 49% (792/1628), between 50–399 copies/ml in 22% (362/1628), between 400–999 copies/ml in 7% (108/1628) and over 1000 copies/ml in 22% (366/1628). Amongst those starting case management with low level viremia (50-999copies/ml), 77% improved from 400–999 copies/ml to a VL below 400copies/ml and 63% improved from 50–399 copies/ml to <50 copies/ml (see Fig 3a).

Amongst clients who did not receive case management post intervention, second VL was suppressed below 50 copies/ml in 44% (710/1628), 25% had a VL between 50–399 copies/ml, 6% had a VL between 400 to 999 copies/ml and 26% had a VL of over 1000 copies/ml. Amongst those who did not receive case management with low level viremia (50-999copies/ml), 81% improved from 400–999 copies/ml to a VL below 400copies/ml and 59% improved from 50–399 copies/ml to <50 copies/ml (see Fig 3b).

Table 3 shows results of the bivariate and multivariable logistic regression analysis of factors associated with viral suppression. In the unadjusted model case management (Odds Ratio [OR] 1.23; Confidence Interval [CI]1.07–1.41) versus no case management, older age groups versus 18–24-year-olds had increased odds of viral suppression. Being male (0.74; 0.64–0.86) had decreased odds of viral suppression.

**Table 3:** Regression of factors associated with viral load suppression stratified by first VL in study.

Variable	Unadjusted regression			Adjusted regression											
	Overall sample N = 3 256			Overall sample N = 3 256			First VL in study <1000 copies/ml N = 1 488			First VL in study 1000 to 9999 copies/ml N = 786			First VL in study >10000 copies/ml N = 982		
	OR	p-value	95% CI	OR	p-value	95% CI	OR	p-value	95% CI	OR	p-value	95% CI	OR	p-value	95% CI
Case management															
No	1.00	.	.	1.00	.	.	1.00	.	.	1.00	.	.	1.00	.	.
Yes	1.23	0.00	1.07–1.41	1.25	0.00	1.08–1.44	1.13	0.26	0.91–1.39	1.50	0.01	1.13–2.01	1.42	0.02	1.06–1.88
Gender															
Female	1.00	.	.	1.00	.	.	1.00	.	.	1.00	.	.	1.00	.	.
Male	0.74	0.00	0.64–0.86	0.72	0.00	0.61–0.84	0.63	0.00	0.50–0.80	1.09	0.60	0.79–1.50	0.81	0.18	0.58–1.10
Age category															
18–24	1.00	.	.	1.00	.	.	1.00	.	.	1.00	.	.	1.00	.	.
25–34	1.38	0.04	1.01–1.89	1.30	0.10	0.95–1.79	1.40	0.21	0.83–2.37	1.38	0.31	0.74–2.56	1.26	0.43	0.71–2.22
35–54	1.45	0.01	1.09–1.94	1.43	0.02	1.07–1.91	1.47	0.11	0.91–2.37	1.41	0.23	0.80–2.48	0.98	0.93	0.58–1.66
55+	1.89	0.00	1.37–2.62	1.88	0.00	1.35–2.61	1.62	0.07	0.97–2.73	1.92	0.05	1.00–3.71	1.19	0.61	0.62–2.26
ART years															
<1 years	1.00	.	.	1.00	.	.	1.00	.	.	1.00	.	.	1.00	.	.
1 to 3 years	0.79	0.17	0.55–1.11	0.79	0.18	0.56–1.11	0.90	0.67	0.54–1.48	0.73	0.45	0.32–1.65	0.72	0.32	0.38–1.38
3 to 10 years	1.00	0.98	0.73–1.35	0.97	0.84	0.71–1.32	1.14	0.58	0.72–1.78	0.99	0.98	0.46–2.11	0.77	0.37	0.43–1.36
over ten years	1.05	0.76	0.75–1.48	1.01	0.94	0.72–1.43	1.29	0.32	0.78–2.15	0.99	0.99	0.44–2.23	0.85	0.63	0.44–1.63

In the adjusted model (Table 3) case management (1.25; 1.08–1.44) versus no case management, 35–54 years old (1.43; 1.07–1.91) and 55+ year old (1.88; 1.35–2.61) versus 18–24-year-old had increased odds of viral suppression whilst being male (0.72; 0.61–0.84) had decreased odds of viral suppression.

When stratified by first VL in study in the adjusted model case management was significantly associated with viral suppression in those with first VL in study of 1000–9999 copies/ml (1.50; 1.13–2.01) or an first VL in study of >10000 copies/ml (1.42; 1.07–1.9) but not a first VL in study of <1000 copies/ml (1.13; 0.91–1.39). Males were less likely to suppress when starting with a first VL in study of <1000 copies/ml (0.63; 0.50–0.80). Those over the age of 55years were more likely to suppress when starting with a first VL in study of <1000 copies/ml (1.62; 0.97–2.73) and 1000 to 9999 copies/ml (1.92; 1.00–3.71) but not >10000 copies/ml.

## Discussion

Our study found that people who enrolled into case management in Capricorn District Limpopo, were more likely to achieve VL suppression (<50 copies/ml) compared to those who didn’t. Despite this improvement, close to half of clients remained unsuppressed, (>50copies/ml), highlighting the need for further intervention to meet the needs of people on ART. Those with a starting VL of >1000 copies/ml were more likely to be suppressed on case management compared to those who did not receive case management. There were no significant associations between viral suppression and case management in the group starting with low-level viremia. To the best of our knowledge this is the first study to assess the effect of case management on PLHIV in routine services in South Africa.

Various studies have shown the effectiveness of enhanced adherence support in improving viral suppression [16–19, 25]. Our results are similar to a Nigerian study, which found a viral suppression rate of 51% after 3 months of EAC support [25] but lower than a South African study conducted in Gauteng Province, where 61% of the participants were virally suppressed after EAC [26]. Similar to our findings, a study conducted in Zimbabwe also found a limited effect of EAC in improving VL [27]. We think it is likely that in our setting with a dolutegravir containing regimen as the preferred first line ART, case management may have decreased some of the barriers to adherence and retention in care, but many barriers still remain [28]. Reasons for poor adherence include individual barriers such as forgetfulness, denial of HIV positive status, side effects and treatment fatigue, as well as fear of stigma and facility levels barriers such as attitudes from health workers and long queues [29–32].

Although poor adherence is the commonest cause of unsuppressed VL, it is important that clients receive appropriate clinical assessment during facility visits to rule out other reasons. Clients remaining unsuppressed after enhanced adherence counselling suggests more complex problems, whether clinical or psychosocial, that require full clinician engagement, additional support, and referrals. Therefore, achieving and maintaining suppression requires a comprehensive approach, beyond adherence counselling, such as differentiated services delivery models, mental health support, or multidisciplinary support. It is important that these additional interventions be strengthened to support those who find it difficult to achieve or maintain suppression. Strong links between retention counsellors and the clinical team are critical so that clients can be assessed and managed holistically. Success in VL suppression also hinges on regular VL monitoring [33, 34]. In our study gaps existed in VL monitoring due to a lack of adherence to guidelines for a repeat VL after 3 months especially in those with a VL between 51–999 copies/ml. Timely VL monitoring is important for the quick identification of virologic failure to prevent drug resistance and improve health outcomes.

Regression analysis showed a statistically significant impact of case management on viral suppression, with those on case management 25% more likely to suppress. When disaggregated by first VL in study we see a higher impact of case management among those with a first VL in study of >1000copies/ml. We speculate that those higher VLs may be prioritized for additional counselling and support within the case management model and by other health care providers.

Our findings also show that being older was associated with higher odds of being virally suppressed whilst men are less likely to suppress reflecting gender disparities within the country and across sub-Saharan Africa [35], with men less likely to access ART [36] or be virally suppressed [37]. Case management needs to ensure it meets the needs of men. The Coach Mpilo peer-support model [38] which is being widely rolled out to provide services similar to case management may assist with this. Differentiated services for virally unsuppressed clients could also be helpful for men.

Our study had some limitations. Our study is not nationally representative as it uses data collected in Capricorn District only. Therefore, the findings may not be generalizable to other districts with different health services or contexts. ART regimen was not included in the control selection and data analysis due to data quality issues. Our study was also limited to adult clients due to the small number of unsuppressed children in the case management sample (n = 222) and because children face different suppression challenges. Therefore, future research is required. Our study used routine data that was collected as part of programme monitoring, making it vulnerable to challenges like missing data. Despite these shortcomings, our study does provide useful information on the effect of a case management intervention in Capricorn. Further qualitative research would complement the quantitative findings from this study to capture the complex nature of factors that influence viral suppression during case management especially at low level viremia.

## Conclusion

Case management had a positive impact on viral suppression in Capricorn District, but more needs to be done to support adherence. Integration of psychosocial and mental health services, continued expansion of differentiated service delivery models, and research into the unmet needs of clients who remain unsuppressed after case management could strengthen adherence. Multidisciplinary teams should work alongside retention counsellors to provide holistic support and ensure that those who remain unsuppressed are receiving enhanced clinical care, EAC and psychosocial support. Additionally, case management should be also strengthened by ensuring the programme meets the needs of men and where possible peer supporters should be used for men and young people.

## Supporting information

S1 Dataset Full dataset of the study. (XLS)

## Abbreviations

ART: Antiretroviral Therapy
EAC: Enhanced Adherence Counselling
HIV: Human Immunodeficiency Virus
PEPFAR: Presidents Emergency Plan for Aids Relief
PLHIV: People Living with HIV
TLD: Tenofovir, Lamivudine and Dolutegravir
USAID: United States Agency for International Development
VL: Viral Load
WHO: World Health Organisation
